# Propofol Reversed Hypoxia-Induced Docetaxel Resistance in Prostate Cancer Cells by Preventing Epithelial–Mesenchymal Transition by Inhibiting Hypoxia-Inducible Factor 1*α*

**DOI:** 10.1155/2018/4174232

**Published:** 2018-01-11

**Authors:** Jiang Qian, Sheliang Shen, Wei Chen, Nianping Chen

**Affiliations:** ^1^Department of Anesthesiology, Zhejiang Hospital, Hangzhou, Zhejiang 310014, China; ^2^Department of Anesthesiology, Zhejiang Provincial People's Hospital, Hangzhou, Zhejiang 310013, China; ^3^Institute of Molecular Engineering, University of Chicago, Chicago, IL 60637, USA; ^4^Department of Anesthesiology, Shaoxing People's Hospital, Shaoxing Hospital of Zhejiang University, Shaoxing, Zhejiang 312000, China

## Abstract

Prostate cancer is the second most frequently diagnosed cancer worldwide. Hypoxia-induced epithelial–mesenchymal transition (EMT), driven by hypoxia-inducible factor 1*α* (HIF-1*α*), is involved in cancer progression and metastasis. The present study was designed to explore the role of propofol in hypoxia-induced resistance of prostate cancer cells to docetaxel. We used the Cell Counting Kit-8 and 5-ethynyl-2′-deoxyuridine incorporation assay to measure cell viability and cell proliferation, respectively, in prostate cancer cell lines. Then, we detected HIF-1*α*, E-cadherin, and vimentin expression using western blotting. Propofol reversed the hypoxia-induced docetaxel resistance in the prostate cancer cell lines. Propofol not only decreased hypoxia-induced HIF-1*α* expression, but also reversed hypoxia-induced EMT by suppressing HIF-1*α*. Furthermore, small interfering RNA–mediated silencing of HIF-1*α* reversed the hypoxia-induced docetaxel resistance, although there was little change in docetaxel sensitivity between the hypoxia group and propofol group. The induction of hypoxia did not affect E-cadherin and vimentin expression, and under the siRNA knockdown conditions, the effects of propofol were obviated. These data support a role for propofol in regulating EMT in prostate cancer cells. Taken together, our findings demonstrate that propofol plays an important role in hypoxia-induced docetaxel sensitivity and EMT in prostate cancer cells and that it is a potential drug for overcoming drug resistance in prostate cancer cells via HIF-1*α* suppression.

## 1. Introduction

Hypoxia is common in the microenvironment of solid tumors and is associated with tumor invasion, distant metastasis, and epithelial–mesenchymal transition (EMT) [1–3]. Hypoxia-inducible factor (HIF) regulates the expression of proteins that increase oxygen delivery, which enables cells to survive in oxygen-deficient conditions [4]. HIF is a heterodimer consisting of the HIF-1*α* and HIF-1*β* transcription factors [5]. HIF-1*α* is the most important hypoxia-induced transcription factor and has multiple functions in tumor progression, including changes in the aggressive behavior of the tumor [6]. Moreover, HIF-1*α* plays a role in prostate cancer cell EMT and migration [7]. EMT is involved in many crucial cancer cell functions, including tissue reorganization, tumorigenesis, cancer recurrence, and metastasis [8]. EMT is characterized by the combined loss of epithelial cell junction proteins such as E-cadherin and the gain of mesenchymal markers such as vimentin or fibronectin [9]. It has become increasingly clear over recent years that EMT, a critical developmental process, plays a major role in cancer progression [10, 11]. Prostate cancer is the most commonly diagnosed malignancy and the second leading cause of cancer death among men in developed countries [12]. Docetaxel is produced semisynthetically from the needles of the Pacific yew tree* (Taxus brevifolia)* [13]. In recent years, docetaxel has been considered standard first-line therapy in prostate cancer cases [14]; however, it confers only a modest survival advantage, as patients eventually acquire docetaxel resistance [15]. However, the mechanisms involved in hypoxia-induced docetaxel resistance remain unclear. Therefore, it is urgent that this mechanism be elucidated.

Propofol (2, 6-diisopropylphenol), a general sedative and hypnotic agent, is widely used for the induction and maintenance of general anesthesia [16]. Accumulating evidence suggests that propofol has several nonanesthetic effects [17]. Recently, it was reported that propofol has potential anticancer properties, such as inhibiting cancer cell proliferation, adhesion, and metastasis and inducing cancer cell apoptosis [18–20]. Recent studies have shown that propofol can suppress cell invasion and reverse EMT by decreasing HIF-1*α* expression in lipopolysaccharide-treated non-small cell lung cancer cells [21]. Furthermore, propofol inhibits viability and induces apoptosis in lung cancer, pancreatic cancer, and cervical cancer cells [22–24]. However, this process has not been completely elucidated in prostate cancer cell lines.

In this study, we found that propofol could reverse hypoxia-induced docetaxel resistance in prostate cancer cells by reversing EMT via HIF-1*α* inhibition. The stronger sensitivity of the cells to the combined docetaxel and propofol treatment as compared with docetaxel-only treatment that we observed shows that propofol sensitized the prostate cancer cells to the hypoxia-induced docetaxel inhibitory effect.

## 2. Materials and Methods

### 2.1. Cell Culture and Induction of Hypoxia

The human prostate cancer cell lines PC3, DU145, and 22RV1 were purchased from American Type Culture Collection (Manassas, VA, USA). All cells were cultured in Roswell Park Memorial Institute (RPMI) 1640 medium (Gibco, Grand Island, NY, USA) supplemented with 10% fetal bovine serum (FBS; Gibco) and 1% penicillin/streptomycin (Sigma-Aldrich, St. Louis, MO, USA). All cells were incubated at 37°C in a humidified atmosphere containing 21% O_2_ and 5% CO_2_. For hypoxic culture, the cells were placed in a hypoxic incubator (1% O_2_, 5% CO_2_) at 37°C for 6 h. HIF-1*α* small interfering RNA (siRNA) and negative siRNA were purchased from Santa Cruz Biotechnology (Dallas, TX, USA). Propofol was purchased from Sigma-Aldrich.

### 2.2. Cell Viability Assay

We used Cell Counting Kit-8 (CCK-8; Dojindo Laboratories, Kumamoto, Japan) to determine the cell viability rate. The cells were seeded in 96-well plates (5 × 10^3^ cells/well) in 100 *μ*L maintenance medium and cultured for 24 h. The culture medium was replaced with 10% FBS–medium containing the drugs (docetaxel (*μ*M): 0, 6.25, 12.5, 25, 50, and 100; propofol (*μ*M): 0, 1.25, 2.5, 5, 10, 20, 40, 80, 160, and 320). After 48-h incubation, 10 *μ*L CCK-8 solution was added, the cells were incubated for 3 h, and then the absorbance at 450 nm was measured using an MRX II microplate reader (Dynex Technologies, Chantilly, VA, USA). The cell viability rate was calculated as a percentage of untreated controls.

### 2.3. HIF-1*α* siRNA Transfection

The cells were seeded in 6-well plates at (1 × 10^5^ cells/well) and transfected with HIF-1*α* siRNA or negative siRNA using Lipofectamine 2000 (Invitrogen, Carlsbad, CA, USA) according to the manufacturer's protocol. The transfection medium (Opti-MEM; Gibco) was removed and replaced with complete medium 6 h after transfection. All experiments were performed for 24 h after transfection and repeated three times.

### 2.4. Western Blot Analysis

Western blotting was used to detect protein expression. Briefly, the cells were lysed with radioimmunoprecipitation assay lysis buffer containing protease inhibitors (Sigma-Aldrich) for 30 min on ice. Then, the lysates were centrifuged at 12000 rpm for 5 min at 4°C. The supernatants were collected and a bicinchoninic acid protein assay kit (Sigma-Aldrich) was used to determine the protein concentrations. Protein (20 *μ*g) from each sample was separated by 10% sodium dodecyl sulfate–polyacrylamide gel electrophoresis and transferred to polyvinylidene difluoride membranes (Millipore, Billerica, MA, USA). The membranes were blocked with 5% bovine serum albumin in Tris-buffered saline and 0.1% Tween 20 (TBST) for 2 h at room temperature and then incubated with primary antibodies (anti–E-cadherin, anti-vimentin, anti–HIF-1*α*, diluted 1 : 1000 in TBST; Abcam, Cambridge, MA, USA) overnight at 4°C. Then, the membranes were washed three times with TBST and incubated with a horseradish peroxidase–conjugated secondary antibody (1 : 2000; Cell Signaling Technology, Beverly, MA, USA) for 2 h at 37°C. *β*-Actin (Cell Signaling Technology) was used as the loading control. Protein bands were detected using an enhanced chemiluminescence detection system (Biological Industries, Beit HaEmek, Israel). Gray value analysis of the protein bands was performed using ImageJ software (National Institutes of Health, Bethesda, MD, USA).

### 2.5. 5-Ethynyl-2′-deoxyuridine (EdU) Analysis

The EdU incorporation assay was used to calculate DNA incorporation/synthesis. Measurement of the rate of cell proliferation inhibition was assessed using a Click-iT EdU Imaging Kit (Thermo Fisher Scientific, Carlsbad, CA, USA) according to a procedure described previously [25].

### 2.6. Statistical Analysis

The experimental data were analyzed using GraphPad Prism 5 software (GraphPad, San Diego, CA, USA) and expressed as the means ± standard deviation (SD). Statistical analysis was conducted using a *t*-test or one- or two-way analysis of variance, followed by Dunnett's or Bonferroni's multiple comparison test. *P* < 0.05 was considered to indicate a statistically significant difference.

## 3. Results

### 3.1. Hypoxia Induced Docetaxel Resistance in Prostate Cancer Cells

**Figure 1:**
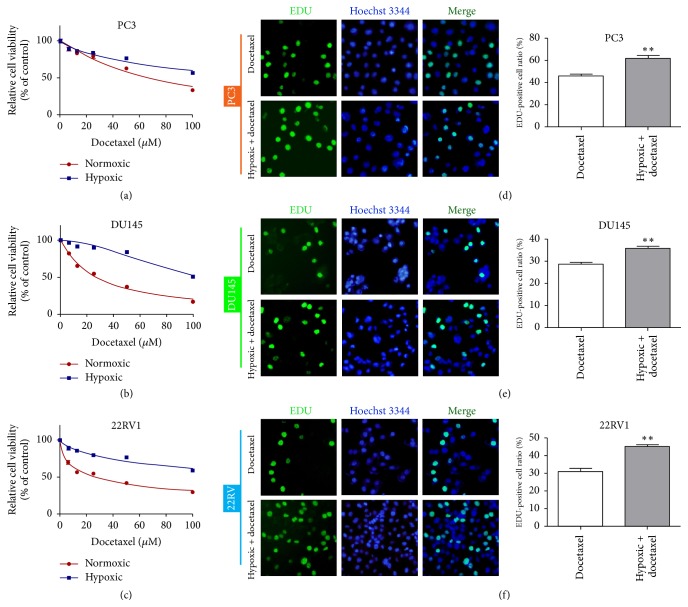
Hypoxia-induced prostate cancer cell resistance to docetaxel. (a–c) CCK-8 detection of viability of prostate cancer cells in normoxic or hypoxic conditions after docetaxel treatment. (d–f) EdU assay detection of the proliferation rate of prostate cancer cells treated with docetaxel under normoxic or hypoxic conditions. ^*∗∗*^*P* < 0.01 versus docetaxel.

To investigate prostate cancer cell sensitivity to docetaxel under normoxic and hypoxic conditions, we used the CCK-8 assay to measure cell viability. Cell viability was significantly increased in hypoxic conditions compared with normoxia (Figures 1(a)–1(c), Table 1), confirming that hypoxia induces docetaxel resistance in prostate cancer cells. The EdU incorporation assay was used to measure the effects of docetaxel on prostate cancer cell proliferation following 48-h treatment under hypoxic conditions. Docetaxel treatment in hypoxia enhanced cell proliferation (*P* < 0.01 versus docetaxel) (Figures 1(d)–1(f)).

### 3.2. Propofol Reversed Hypoxia-Induced Docetaxel Resistance in Prostate Cancer Cells

**Figure 2:**
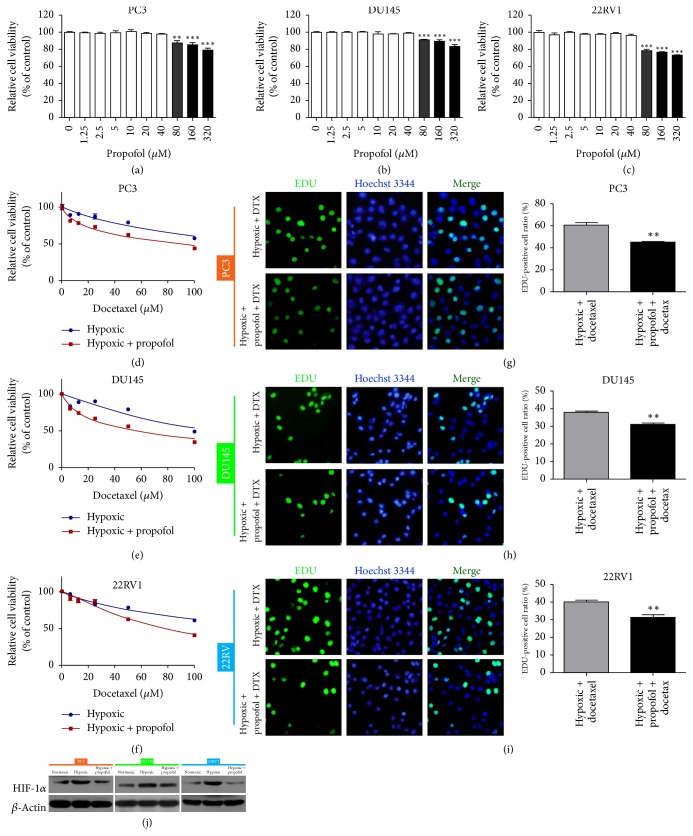
Propofol reversed hypoxia-induced docetaxel resistance in prostate cancer cells. (a–c) Human prostate cancer cells were treated with propofol for 48 h, and cell viability was measured using CCK-8. ^*∗∗∗*^*P* < 0.001 versus 0 *μ*M. (d–f) Cell viability was measured in prostate cancer cells treated with docetaxel with or without propofol (40 *μ*M) under hypoxic conditions for 48 h. (g–i) EdU assay detection of the proliferation rate of prostate cancer cells treated with docetaxel or docetaxel plus propofol under hypoxic conditions. Histograms represent the positive cell rate (%). ^*∗∗*^*P* < 0.01 versus hypoxia + docetaxel. DTX, docetaxel. (j) Western blotting detection of HIF-1*α* expression following propofol treatment under hypoxic or normoxic conditions.

To evaluate whether propofol could reverse hypoxia-induced docetaxel resistance, we used CCK-8 to measure the viability of cells treated with docetaxel alone or with docetaxel combined with propofol under hypoxic conditions. Initially, 80, 160, or 320 *μ*M propofol significantly inhibited cell viability compared with 0 *μ*M propofol (Figures 2(a)–2(c)). Then, we selected the highest concentration of propofol (40 *μ*M) that caused little effect for further analyses. Surprisingly, docetaxel sensitivity was enhanced after combination with propofol (Figures 2(d)–2(f), Table 2). The EdU incorporation assay indicated that the EdU-positive cell ratio was significantly decreased compared to the hypoxia + docetaxel group (*P* < 0.01) (Figures 2(g)–2(i)). Then we detected HIF-1*α* expression in prostate cancer cells under hypoxic conditions and with or without propofol and found that hypoxia induced HIF-1*α* upregulation, whereas propofol suppression of HIF-1*α* expression reversed this result (Figure 2(j), Table 3). These findings demonstrate that propofol reverses hypoxia-induced docetaxel resistance in prostate cancer cells.

### 3.3. Propofol Partially Reversed Hypoxia-Induced EMT in Prostate Cancer Cells

**Figure 3:**
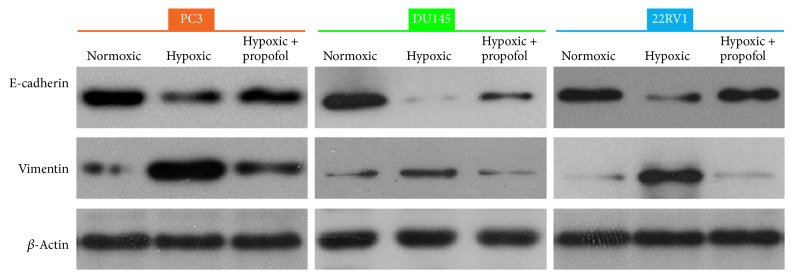
Propofol partially reversed hypoxia-induced EMT. Western blot showing E-cadherin and vimentin expression in prostate cancer cells treated with or without propofol under hypoxic or normoxic conditions.

To determine whether the mechanism of propofol reversed hypoxia-induced docetaxel resistance is related to EMT, we detected E-cadherin and vimentin expression by western blotting. Hypoxia downregulated E-cadherin expression and increased vimentin expression in the cells. In addition, E-cadherin expression was increased and vimentin expression was decreased after propofol treatment as compared with the hypoxia-induced group, indicating that propofol can reverse hypoxia-induced EMT in prostate cancer cells (Figure 3, Tables 4 and 5).

### 3.4. HIF-1*α* Knockdown Partially Reversed Hypoxia-Induced Docetaxel Resistance

**Figure 4:**
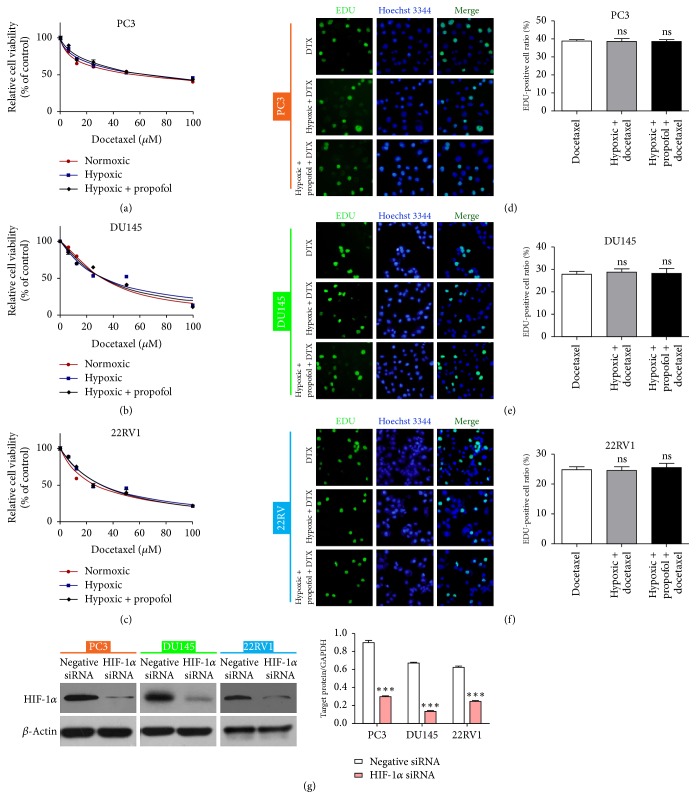
HIF-1*α* knockdown partially reversed hypoxia-induced docetaxel resistance. (a–c) Cell viability assay quantification of prostate cancer cells treated with docetaxel with or without propofol under normoxic or hypoxic conditions following HIF-1*α* knockdown. (d–f) EdU assay detection of the proliferation rate of prostate cancer cells following HIF-1*α* knockdown and treatment with docetaxel, docetaxel plus propofol in hypoxia, or docetaxel alone in normoxia. (g) Western blot assessment of HIF-1*α* knockdown efficiency in hypoxia condition; histogram represents the average grey value of the HIF-1*α* protein. ^*∗∗∗*^*P* < 0.001 versus control. DTX, docetaxel; ns, not significant.

Hypoxia promotes EMT by activating HIF-1*α* [26]. Although we had determined that propofol could reverse hypoxia-induced EMT in prostate cancer cells, it was unclear whether the effect of propofol was related to HIF-1*α*. We hypothesized that propofol affects hypoxia-induced docetaxel resistance in prostate cancer cells by regulating HIF-1*α*. To further examine this effect, we transfected HIF-1*α* siRNA into prostate cancer cells and then examined their viability following docetaxel treatment with or without propofol in hypoxic conditions or with docetaxel alone in normoxia; there was little change in the docetaxel resistance among the three groups (Figures 4(a)–4(c), Table 6). Furthermore, HIF-1*α* knockdown partially reversed the hypoxia-induced docetaxel resistance (Figures 4(a)–4(c)). The proliferation-suppressing effect of docetaxel was verified by the EdU assay, although there was no significant difference among the groups (Figures 4(d)–4(f)). Western blotting was performed to determine knockdown efficiency (Figure 4(g)). The findings show that inhibiting HIF-1*α* reverses hypoxia-induced docetaxel resistance in PC cells.

### 3.5. Propofol Played a Role in Prostate Cancer Cell Docetaxel Sensitivity by Decreasing HIF-1*α*

**Figure 5:**
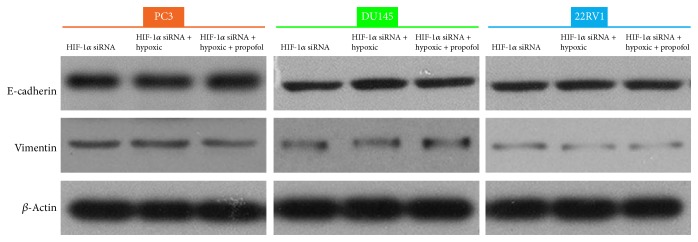
Propofol played a role in hypoxia-induced docetaxel resistance by decreasing HIF-1*α*. Western blot showing E-cadherin and vimentin expression in HIF-1*α*–knockdown prostate cancer cells under hypoxia treated with or without propofol.

We determined that hypoxia induced E-cadherin downregulation and vimentin upregulation. To explore the mechanism underlying EMT and hypoxia-induced docetaxel resistance due to HIF-1*α* overexpression in prostate cancer cells, we knocked down HIF-1*α* and examined E-cadherin and vimentin expression using western blotting. Interestingly, hypoxia did not affect E-cadherin and vimentin expression. However, the effects of propofol were blocked such that there was no significant change in the levels of expression of EMT markers between any of the groups (Figure 5(a), Tables 7 and 8).

## 4. Discussion

Docetaxel is more effective against progressive human prostate cancer than other conventional anticancer agents [27]. Unfortunately, drug resistance negatively impacts the effects of this treatment. The mechanisms involved in docetaxel resistance in hypoxia remain unclear, so further studies are needed to clarify this issue.

In clinical practice, propofol is commonly used in the induction and maintenance of general anesthesia. An intravenously administered hypnotic agent, propofol, is widely used in all types of surgeries due to its short effect and rapid recovery. A variety of studies have illustrated its neuroprotective property [28, 29]. Recently, research has focused on its antitumor effect [30]. Several studies have shown that propofol can be used in combination with current clinical chemotherapeutic drug regimens such as gemcitabine or paclitaxel [31, 32]. Moreover, it had been proved that propofol works as an antioxidant and that propofol can act in an antioxidant capacity mainly on mitochondrial Complex I to decrease cellular ROS levels required to stabilize HIF [33]. In the present study, we hypothesized that propofol is involved in docetaxel resistance in prostate cancer cells. Western blotting and CCK-8 showed that hypoxia induced docetaxel resistance in prostate cancer cells, which is consistent with that reported for breast cancer cells in response to doxorubicin [34]. Docetaxel treatment in hypoxic condition enhanced prostate cancer cell proliferation. However, combining docetaxel with propofol enhanced docetaxel sensitivity in prostate cancer cells.

EMT is considered an essential step in cancer progression and metastasis because it allows cancer cells to migrate, invade the surrounding tissues, and escape into the bloodstream, such that primary tumors can metastasize to other organs [35]. Some microenvironmental factors, such as hypoxia, are also involved in EMT during malignant cell transformation [36]. Hypoxia-induced HIF-1*α* expression enhances EMT and induces resistance to radiotherapy and chemotherapy, promoting tumor migration and invasion. EMT may be a key process regulating resistance to chemotherapy in malignant tumors [37, 38]. NSCLC cells with an epithelial phenotype are more sensitive to chemotherapy than those with a mesenchymal phenotype. We proved that combined docetaxel with propofol enhanced sensitivity under hypoxia condition in prostate cancer cells, and it was related to HIF-1*α* expression. Then western blot analysis showed that hypoxia induced HIF-1*α* upregulation compared with normoxia, but propofol induced the HIF-1*α* downregulation as compared with hypoxia. Next, we attempted to identify whether propofol has EMT-inhibiting capability. We directly evaluated the effect of propofol on several hypoxia-mediated EMT parameters, including E-cadherin and vimentin expression levels. We found that propofol inhibited the hypoxia-induced EMT and reversed the hypoxia-induced E-cadherin downregulation and vimentin upregulation. Taken together, these data indicate that propofol reverses hypoxia-induced docetaxel resistance in prostate cancer cells by preventing EMT. HIF-1*α* is a key transcription factor induced by hypoxia [39] and it activates the transcription of genes implicated in tumor angiogenesis, cell survival, and resistance to chemotherapeutic drugs [40–42]. Thus, inhibiting HIF-*α* activity or EMT might be a new target for innovative mechanism-based drug discovery for cancer disease. We transfected HIF-1*α* siRNA into prostate cancer cells to knock down HIF-1*α* expression and found that hypoxia-induced docetaxel resistance disappeared and that propofol did not reverse docetaxel resistance in prostate cancer cells after HIF-1*α* siRNA transfection.

In conclusion, we have established that propofol plays an important role in hypoxia-induced docetaxel resistance in prostate cancer cells. Propofol reversed hypoxia-induced docetaxel resistance through EMT by inhibiting hypoxia-induced HIF-1*α* expression. These results indicate that propofol has potential as a therapeutic agent for improving prostate cancer treatment.

## Figures and Tables

**Table 1:** The cell viability of PC cells treated with different concentrations of docetaxel under normoxia and hypoxia conditions.

Cell lines	IC_50_ (*μ*M)	
	Normoxic	Hypoxic
PC3	66.26 (58.30 to 74.22)	169.6 (123.9 to 215.2)
DU145	27.03 (25.46 to 28.59)	106.9 (97.00 to 116.8)
22RV1	26.73 (22.92 to 30.54)	208.7 (130.2 to 287.2)

IC_50_ values show docetaxel concentration [*μ*M, mean (95% confidence intervals)].

**Table 2:** The cell viability of PC cells treated with docetaxel alone or docetaxel plus propofol under hypoxia conditions.

Cell lines	IC_50_ (*μ*M)	
	Hypoxic	Hypoxic + propofol
PC3	159.1 (107.6 to 210.6)	88.00 (69.51 to 106.5)
DU145	114.4 (86.92 to 141.9)	54.45 (48.74 to 60.16)
22RV1	179.4 (131.1 to 227.6)	77.67 (67.55 to 87.79)

IC_50_ values show docetaxel concentration [*μ*M, mean (95% confidence intervals)].

**Table 3:** The grey value of the HIF-1*α* protein treated with or without propofol under normoxia or hypoxia conditions.

Cell lines	HIF-1*α*/*β*-actin		
	Normoxic	Hypoxic	Hypoxic + Propofol
PC3	0.448049	0.942486	0.508608
DU145	0.420623	1.034483	0.698514
22RV1	0.439184	1.303041	0.549622

**Table 4:** The grey value of the E-cadherin protein treated with or without propofol under normoxia or hypoxia conditions.

Cell lines	E-cadherin/*β*-actin		
	Normoxic	Hypoxic	Hypoxic + propofol
PC3	1.167701	0.66641	0.906073
DU145	0.972721	0.038215	0.421248
22RV1	0.641246	0.174243	0.555517

**Table 5:** The grey value of the vimentin protein treated with or without propofol under normoxia or hypoxia conditions.

Cell lines	Vimentin/*β*-actin		
	Normoxic	Hypoxic	Hypoxic + propofol
PC3	0.2797	1.436738	0.685475
DU145	0.128068	0.335682	0.154392
22RV1	0.071099	0.741434	0.109708

**Table 6:** The cell viability of PC cells treated with docetaxel alone under normoxia or hypoxia conditions or docetaxel plus propofol under hypoxia conditions.

Cell lines	IC_50_ (*μ*M)		
	Normoxic	Hypoxic	Hypoxic + propofol
PC3	55.73 (46.25 to 65.21)	64.58 (54.11 to 75.05)	65.54 (52.64 to 78.43)
DU145	31.44 (29.31 to 33.58)	32.20 (27.81 to 36.59)	31.63 (28.10 to 35.17)
22RV1	26.61 (23.49 to 29.73)	31.65 (27.96 to 35.35)	31.10 (27.38 to 34.81)

IC_50_ values show docetaxel concentration [*μ*M, mean (95% confidence intervals)].

**Table 7:** The grey value of the E-cadherin protein treated with or without propofol after knockdown of HIF-1*α* under normoxia or hypoxia conditions.

Cell lines	E-cadherin/*β*-actin		
	HIF-1*α* siRNA	HIF-1*α* siRNA + hypoxic	HIF-1*α* siRNA + hypoxic + propofol
PC3	0.66447	0.64527	0.675951
DU145	0.431083	0.4735	0.443874
22RV1	0.426146	0.465596	0.447101

**Table 8:** The grey value of the vimentin protein treated with or without propofol after knockdown of HIF-1*α* under normoxia or hypoxia conditions.

Cell lines	Vimentin/*β*-actin		
	HIF-1*α* siRNA	HIF-1*α* siRNA + hypoxic	HIF-1*α* siRNA + hypoxic + propofol
PC3	0.144313	0.152617	0.154287
DU145	0.109956	0.107534	0.105961
22RV1	0.049789	0.048136	0.040924
